# AI-Generated Information for Vascular Patients: Assessing the Standard of Procedure-Specific Information Provided by the ChatGPT AI-Language Model

**DOI:** 10.7759/cureus.49764

**Published:** 2023-11-30

**Authors:** Omar Haidar, Alexander Jaques, Pierre W McCaughran, Matthew J Metcalfe

**Affiliations:** 1 Vascular Surgery, Lister Hospital, Stevenage, GBR

**Keywords:** chatgpt, patient education, ai, artificial intelligence, vascular

## Abstract

Introduction

Ensuring access to high-quality information is paramount to facilitating informed surgical decision-making. The use of the internet to access health-related information is increasing, along with the growing prevalence of AI language models such as ChatGPT. We aim to assess the standard of AI-generated patient-facing information through a qualitative analysis of its readability and quality.

Materials and methods

We performed a retrospective qualitative analysis of information regarding three common vascular procedures: endovascular aortic repair (EVAR), endovenous laser ablation (EVLA), and femoro-popliteal bypass (FPBP). The ChatGPT responses were compared to patient information leaflets provided by the vascular charity, Circulation Foundation UK. Readability was assessed using four readability scores: the Flesch-Kincaid reading ease (FKRE) score, the Flesch-Kincaid grade level (FKGL), the Gunning fog score (GFS), and the simple measure of gobbledygook (SMOG) index. Quality was assessed using the DISCERN tool by two independent assessors.

Results

The mean FKRE score was 33.3, compared to 59.1 for the information provided by the Circulation Foundation (SD=14.5, p=0.025) indicating poor readability of AI-generated information. The FFKGL indicated that the expected grade of students likely to read and understand ChatGPT responses was consistently higher than compared to information leaflets at 12.7 vs. 9.4 (SD=1.9, p=0.002). Two metrics measure readability in terms of the number of years of education required to understand a piece of writing: the GFS and SMOG. Both scores indicated that AI-generated answers were less accessible. The GFS for ChatGPT-provided information was 16.7 years versus 12.8 years for the leaflets (SD=2.2, p=0.002) and the SMOG index scores were 12.2 and 9.4 years for ChatGPT and the patient information leaflets, respectively (SD=1.7, p=0.001). The DISCERN scores were consistently higher in human-generated patient information leaflets compared to AI-generated information across all procedures; the mean score for the information provided by ChatGPT was 50.3 vs. 56.0 for the Circulation Foundation information leaflets (SD=3.38, p<0.001).

Conclusion

We concluded that AI-generated information about vascular surgical procedures is currently poor in both the readability of text and the quality of information. Patients should be directed to reputable, human-generated information sources from trusted professional bodies to supplement direct education from the clinician during the pre-procedure consultation process.

## Introduction

As we progress through the ‘information age’, there is an increasing number of sources through which patients can learn about their pathology and the treatment options available to them. Despite this volume increasing, there can be no assurances as to its quality. Large variances exist in both the form and content of patient-facing educational material [1]. Ensuring access to high-quality information is paramount to facilitating informed surgical decision-making for patients undergoing vascular procedures.

Artificial intelligence

One increasingly popular method of gathering information is through the utilization of artificial intelligence (AI). ChatGPT is an AI large language model (LLM) created by OpenAI (San Francisco, CA, USA) [2], which has been trained with information input from a wide variety of publicly available sources, including books, articles, and websites. Once a query is inputted, ChatGPT uses its language model to organize this data into a coherent, grammatical, and contextually correct answer. Since its release in November 2022, it has been reported to have received over 1.5 billion page visits [3]. The use of AI is established in many consumer-facing roles, i.e., customer support or virtual assistance, and is increasingly being deployed in the healthcare sector. In 2023, the UK government announced £100 million into the *AI Life Sciences Accelerator Mission* to utilize AI in the National Health Service [4].

Research has been completed on the possible uses of ChatGPT for the vascular clinician. In a comprehensive literature review, Fischer et al. explored the uses of AI and machine learning in vascular diagnostics, perioperative risk stratification, and outcome prediction [5]. The technology is still relatively nascent in these fields, requiring further clinical validation and integration into healthcare systems. Our search found no literature on the use of AI in patient health information acquisition.

Patient education

The ultimate responsibility for ensuring adequately informed patient decision-making lies with the consenting clinician per the Royal College of Surgeons of England’s (RCSE) Good Surgical Practice guidelines on consent [6]. Supplementary materials have long been used to facilitate patient education, namely information leaflets, educational videos, and increasingly, webpages and internet resources. Their use is explicitly endorsed in the RCSE guidelines. Artificial intelligence language models also have the potential to educate, whether the patient is directed there by the clinician or not.

The quality of information available on the internet to educate vascular patients was first investigated in 1999 by Soot et al., who analyzed the 50 most common websites encountered when searching for common vascular pathologies on search engines. They remarked that “overall quality is poor, and information is difficult to obtain in part because of the large number of irrelevant sites” [7]. The question was revisited in 2012 by Grewal et al., who repeated a similar study, analyzing common websites encountered by major search engines [8]. They analyzed readability through the FKRE score, the Gunning fog index, and the LIDA tool to assess reliability; they concluded that “internet information on vascular surgical conditions and procedures is poorly written and unreliable,” indicating little progression 13 years on. As online resources have developed a decade on from Grewal et al.’s study, and with the recent introduction of AI language models claiming to be able to present this growing information pool in a readable and conversational manner, the question of the internet’s ability to inform the vascular patient population is worth revisiting.

## Materials and methods

We performed a retrospective qualitative assessment of documents intended for patient use, comparing those generated by AI and those produced by human writing. The Circulation Foundation is a UK-registered charity (Charity Number 1102769) and a division of the United Kingdom’s Vascular Society [9]. Patient information leaflets were accessed in July 2023 regarding three commonly performed vascular procedures covering domains of aortic, peripheral artery, and venous pathology. These were endovascular aortic repair (EVAR) [10], femoro-popliteal bypass (FPBP) [11], and endovenous laser therapy (EVLA) [12]. ChatGPT, the AI LLM developed by OpenAI using the GPT-3.5 framework [2], was also accessed in the same month, i.e., July 2023. The input questions were as follows: “I am a patient who has been offered [Vascular Procedure]. What should I know before agreeing to have this procedure?” Responses were then extracted (see Appendix A-C) and assessed for readability and quality.

Assessments of standard

Readability was assessed using the FKRE score, FKGL, GFS, and the SMOG index. Scores were derived from the WebFX (Harrisburg, PA, USA) online readability test [13]. Data is presented as raw values and means. The FKRE score is given on a scale of 0-100, with higher scores signifying better readability. Score creators recommend a target score of 65 or higher. The FKGL is an estimation of the education grade (as per the American education system) required to understand a piece of writing. A lower score indicates more readily accessible information. A recommended target for general audiences is 7th grade or lower. The GFS and SMOG are both measures referring to the years of education required to comprehend material. Once again, a lower score indicates better readability.

Quality assessments were performed using the DISCERN tool by two independent assessors (AJ and PM). Scores are presented as raw scores out of 80, and means were also calculated. Inter-rater reliability was assessed using Cronbach’s alpha score of reliability.

Normality was assessed using the Kolmogorov-Smirnov test of normality (see Appendix D). The data was found to be normally distributed, so parametric tests were used. Graphing and statistical analysis were performed in Microsoft Excel (Microsoft Corp., Redmond, WA, USA).

## Results

Readability metrics for AI-generated answers were low across all procedures (Table 1). The mean FKRE score was 33.3, compared to 59.1 for the information provided by the Circulation Foundation (SD=14.5, p=0.025). No information surpassed the benchmark score of 65 as detailed by the metric creators as a good level for the general population. The FKGL indicated that the expected grade of students likely to read and understand ChatGPT responses was consistently higher than compared to information leaflets at 12.7 vs. 9.4 (SD=1.9, p=0.002). Two metrics that measured readability in terms of the number of years of education required to understand a piece of writing were the GFS and SMOG. Both scores indicated that AI-generated answers were less accessible. The GFS for ChatGPT-provided information was 16.7 years vs. 12.8 years for the leaflets (SD=2.2, p=0.002); and SMOG index scores were 12.2 and 9.4 years for ChatGPT and patient information leaflets, respectively (SD=1.7, p=0.001).

**Table 1:** Readability scores for all patient information documents FKRE: Flesch-Kincaid reading ease score, FKGL: Flesch-Kincaid grade level, GFS: Gunning fog score, SMOG: Simple measure of gobbledygook, EVAR: Endovascular aortic repair, EVLA: Endovenous laser ablation, FPBP: Femoro-popliteal bypass

Readability scores	ChatGPT				Patient information leaflet			
	EVAR	EVLA	FPBP	Mean	EVAR	EVLA	FPBP	Mean
FKRE	37.6	31.9	30.5	33.3	55.1	61.5	60.6	59.1
FKGL	12.8	12.5	12.9	12.7	10.4	8.7	9.2	9.4
GFS	16.7	16.5	16.8	16.7	13.8	11.6	12.9	12.8
SMOG	12.5	12	12.2	12.2	10.2	8.5	9.4	9.4

The content of AI-generated information was of lower quality globally. The Cronbach alpha inter-observer reliability score was 0.9795, indicating excellent reliability of DISCERN scores between assessors. The DISCERN scores were consistently higher in human-generated patient information leaflets compared to AI-generated information across all procedures (Table 2). Scores are out of a possible 80. The mean score for the information provided by ChatGPT was 50.3 vs. 56.0 for the Circulation Foundation information leaflets (SD=3.38, p<0.001).

**Table 2:** The DISCERN tool scores for all patient information documents EVAR: Endovascular aortic repair, EVLA: Endovenous laser ablation, FPBP: Femoro-popliteal bypass

DISCERN tool score	ChatGPT				Patient information leaflet			
	EVAR	EVLA	FPBP	Mean	EVAR	EVLA	FPBP	Mean
	49	52.5	49.5	50.3	55	58	55	56.0

## Discussion

Almost a quarter century after Soot et al.’s initial investigation of internet resources for vascular patient education, our analysis found that, despite the introduction of AI boasting accurate and conversational information, there is much progress needed before it can be endorsed for this purpose. When compared to the benchmark of information written by humans and tailored to vascular patient audiences, ChatGPT responses were inferior both in readability and quality of information. Clinicians should continue to direct patients to these resources and remember that the ultimate responsibility for educating patients during the consenting process lies with them.

Material risks

Beyond discussions regarding the quality of information provided by AI, it is important at this stage to recall the landmark case of Montgomery vs. Lanarkshire Health Board [14], which defined a duty of care on clinicians to make patients aware of all *material risks*. The case defined materiality as “a reasonable person in the patient’s position would be likely to attach significance to the risk, or the doctor is or should reasonably be aware that the particular patient would be likely to attach significance to it” [14]. Focusing on the latter half of this statement, we are reminded that direct clinician input is required to tailor all education to the particular situation of the patient. The distribution of informational resources is no replacement for direct clinician input, but they are there to be used as a reminder and supplemental reading for the patient.

Guidelines for use

The RCSE details guidelines on consent in Section 3.5.1 of Good Surgical Practice [6]. It states:

“Where possible, you should provide written information to patients to enable them to reflect and confirm their decision. You should also provide advice on how they can obtain further information to understand the procedure and their condition. This can include information such as patient leaflets, decision aids, websites and educational videos.”

Our study recommends that, while adhering to these guidelines, vascular surgeons should not be directing patients to AI LLMs for further information. As detailed by the RCSE, patient leaflets from reputable professional bodies should remain the standard for supplementary patient education. Currently, no guidelines are readily available for the use of AI in surgery from any UK professional surgical body; however, these issues span beyond just the field of vascular surgery. Similar poor outcomes for readability were found in Momenaei et al.'s study assessing AI-generated information on retinal disease [15] and Musheyev et al.'s study regarding urological malignancies [16].

Interestingly, both studies found the information to be accurate and appropriate in content, which was not reproduced in our findings in vascular surgery. This discrepancy indicates potential specialty-dependent inconsistencies in the quality of data used to inform answers. We recommend further investigation into the current use of AI by clinicians and patients across specialties and to dissuade their further use until the quality of the information can be reliably and reproducibly assured. Additionally, given that this difference exists between surgical specialties, it may also exist between subjects within the same field. Further research is required to determine inter- and intra-specialty differences in the accuracy of AI-generated information.

Quality assurance

The topic of data quality from AI was discussed at the National Institute of Health workshop held in 2019 [17]. The panel concluded that the trustworthiness of AI in healthcare is currently impeded by data quality; this finding is reproduced in our analysis. If AI is to be integrated into the domain of patient education, further clinical input should be sought to define the data sets that AI uses to source information as well as quality assurance for patient-facing output. We echo the call from Park et al. for further clinical validation through a study into the current state of AI in healthcare [18]. Just as with any tool or intervention, research must be conducted to determine the validity of AI-generated information before its endorsed integration into health systems, and repeated quality assurance must be undertaken as these systems evolve.

Limitations

This study utilized ChatGPT based on the GPT-3.5 framework. OpenAI has released GPT-4 and integrated a newer iteration. However, this is restricted behind a paywall of $20 per month as part of ChatGPT Plus [19]. OpenAI reports that GPT-4 is “40% more likely to produce factual responses than GPT-3.5 on our internal evaluations" [19]. We decided to use the free-to-use version that is more readily accessible to patients and therefore more likely to represent the experience of patients turning to LLM’s to research proposed vascular procedures. Similarly, within the same iteration of ChatGPT, answers may contain slight differences when asked on different occasions. Scores may have differed if queries were requested at different times.

The formulae of the four readability metrics used commonly rely on the same two parameters in their assessments, i.e., syllables per word and words per sentence. While providing an objective metric that is useful to facilitate direct comparison, it cannot always be assumed that shorter words are simpler to comprehend. This highlights an important distinction to note between the readability and understandability of language [20]. Outside of formulaic approaches, eye-movement tracking is an alternative proposed method of readability assessment [21] and is an exciting avenue for potential further study to assess the reproducibility of findings.

## Conclusions

Artificial intelligence-generated information was found to be comparatively poor in both readability and quality when compared to existing educational material from human experts. Vascular clinicians should favor patient information leaflets from reputable professional institutions when directing patients to supplementary educational material. Further regulation is required to standardize the data input into AI language models and audit the quality of patient-facing output before it can be utilized safely and effectively as an educational resource.

## Appendices

Appendix A 

ChatGPT Information on EVAR

Prompt: 

I am a patient who has been offered Endovascular Aortic Aneurysm Repair. What should I know before agreeing to have this procedure?

Answer:

As an AI language model, I can provide you with some general information about Endovascular Aortic Aneurysm Repair (EVAR) to help you understand the procedure. However, please note that I'm not a doctor, so it's important to consult with a qualified healthcare professional who can provide you with personalized medical advice based on your specific condition. Here are some key points to consider and discuss with your healthcare provider: 1. What is Endovascular Aortic Aneurysm Repair (EVAR)?   - EVAR is a minimally invasive procedure used to treat an aortic aneurysm, which is a bulging or weakening of the wall of the aorta (the main blood vessel that carries blood from the heart to the rest of the body).   - During EVAR, a stent graft (a fabric tube supported by a metal frame) is inserted into the weakened area of the aorta to reinforce it and prevent the aneurysm from rupturing. 2. Suitability for EVAR:   - Not all aortic aneurysms are suitable for EVAR. Factors such as the size, shape, and location of the aneurysm, as well as the condition of the blood vessels, need to be evaluated to determine if you are a candidate for the procedure.   - Your healthcare provider will assess your medical history, perform imaging tests (such as CT scans or ultrasound), and consider other factors before recommending EVAR. 3. Benefits of EVAR:   - EVAR is a less invasive alternative to traditional open surgery for aortic aneurysm repair.   - The procedure is associated with a shorter hospital stay, reduced recovery time, and less post-operative pain compared to open surgery.   - The risk of complications, such as infection and blood loss, may be lower with EVAR. 4. Potential risks and complications:   - While EVAR generally has a lower risk profile than open surgery, there are still potential risks and complications involved.   - These may include injury to blood vessels during the procedure, leakage around the stent graft (endoleak), infection, blood clots, aneurysm rupture, and the need for additional procedures or interventions.   - Long-term follow-up is usually required to monitor the durability of the stent graft and identify any potential complications that may arise over time. 5. Alternatives and considerations:   - Open surgical repair: Discuss with your healthcare provider the possibility of open surgery as an alternative to EVAR. They can explain the pros and cons of each approach based on your specific situation.   - Lifestyle modifications: Depending on the size and progression of the aneurysm, your healthcare provider may recommend monitoring it regularly through imaging tests and making lifestyle changes to manage risk factors such as high blood pressure and smoking. 6. Post-operative care and follow-up:   - It's important to understand the post-operative care instructions and follow them diligently.   - You may need regular follow-up appointments and imaging tests to monitor the success of the procedure and detect any potential complications. Remember, this information is a general overview, and your healthcare provider will provide you with more detailed information based on your specific condition. It's essential to have an open and thorough discussion with your doctor to ensure you fully understand the procedure, risks, benefits, and any alternatives available to you.

Appendix B

ChatGPT Information on EVLA

Prompt:

I am a patient who has been offered Endovenous Laser Ablation Therapy. What should I know before agreeing to have this procedure?

Answer: 

Endovenous Laser Ablation (EVLA) therapy is a minimally invasive procedure used to treat varicose veins. Before agreeing to have this procedure, there are several important factors to consider. Here's what you should know: 1. Varicose Vein Evaluation: It's crucial to undergo a thorough evaluation by a qualified healthcare professional, usually a vascular specialist or a vascular surgeon, who can assess the severity of your varicose veins and determine if EVLA is the appropriate treatment for your condition. They will examine your medical history, perform a physical examination, and may order diagnostic tests like ultrasound to evaluate the veins. 2. Procedure Overview: EVLA involves the use of laser energy to close off and seal abnormal veins. During the procedure, a thin laser fiber is inserted into the affected vein through a small incision. The laser emits heat, which causes the vein to collapse and seal shut. Blood flow is then naturally rerouted to healthier veins. 3. Benefits: EVLA offers several potential benefits, including improved cosmetic appearance by reducing the appearance of varicose veins, relief from symptoms like pain, swelling, and leg heaviness, and potential prevention of complications associated with varicose veins such as ulcers and blood clots. 4. Risks and Complications: While EVLA is generally safe, it's important to be aware of potential risks and complications. These can include temporary bruising, swelling, numbness, or pain in the treated area. In rare cases, infection, blood clot formation, nerve injury, skin burns, or deep vein thrombosis (DVT) may occur. 5. Recovery and Downtime: The recovery period after EVLA is usually shorter compared to traditional vein stripping surgery. You may be able to resume normal activities within a day or two, although it's recommended to avoid strenuous activities and heavy lifting for a few weeks. Your healthcare provider will provide specific post-procedure instructions. 6. Alternatives and Combination Treatments: EVLA may not be suitable for everyone, and in some cases, alternative treatments or a combination of therapies may be recommended. Other treatment options for varicose veins include sclerotherapy, radiofrequency ablation, and ambulatory phlebectomy. Discuss these alternatives with your healthcare provider to determine the best approach for your specific situation. 7. Success Rates and Long-Term Outcomes: EVLA has shown high success rates in treating varicose veins, with improvement in symptoms and cosmetic appearance. However, it's essential to have realistic expectations. Some patients may require multiple sessions or additional treatments to achieve optimal results. Varicose veins can recur over time, especially if underlying factors like obesity or prolonged standing are not addressed. 8. Cost and Insurance Coverage: Check with your healthcare provider and insurance company regarding the cost of EVLA therapy and whether it's covered by your insurance plan. Insurance coverage can vary, and you may need pre-authorization or meet specific criteria for reimbursement. Remember to have a thorough discussion with your healthcare provider, ask questions, and address any concerns you may have before making an informed decision about undergoing Endovenous Laser Ablation therapy.

Appendix C

ChatGPT Information on FPBP 

Prompt:

I am a patient who has been offered Femoro-popliteal Bypass Surgery What should I know before agreeing to have this procedure?

Answer:

Femoro-popliteal bypass surgery, also known as lower extremity bypass surgery, is a surgical procedure performed to improve blood flow in the lower limbs by bypassing blocked or narrowed arteries. Before agreeing to have this procedure, there are several important things you should know: 1. Diagnosis and indications: Understand why your doctor is recommending femoro-popliteal bypass surgery. This procedure is typically recommended for patients with peripheral arterial disease (PAD) or severe peripheral artery occlusive disease (PAOD) who experience symptoms such as leg pain, non-healing wounds, or decreased mobility due to reduced blood flow. 2. Risks and complications: Like any surgical procedure, femoro-popliteal bypass surgery carries certain risks and potential complications. These can include infection, bleeding, blood clots, graft failure, nerve injury, and heart or lung problems. Your doctor should discuss these risks with you and address any concerns you may have. 3. Alternatives: It's important to explore alternative treatment options before opting for surgery. Depending on your condition, these alternatives may include lifestyle modifications (such as quitting smoking, exercise, and dietary changes), medication management, or minimally invasive procedures like angioplasty or stenting. 4. Preoperative preparation: Prior to the surgery, your healthcare team will guide you through preoperative instructions. This may include fasting for a certain period before the procedure, stopping certain medications or supplements, and providing information about any allergies or medical conditions you may have. 5. Anesthesia: Femoro-popliteal bypass surgery is usually performed under general anesthesia, meaning you will be unconscious during the procedure. An anesthesiologist will be responsible for administering and monitoring the anesthesia to ensure your safety. 6. Hospital stay and recovery: Following the surgery, you will typically be required to stay in the hospital for a few days. Your healthcare team will monitor your progress, manage pain, and provide postoperative care instructions. You may need to wear compression stockings and take medication to prevent blood clots. A rehabilitation program might be recommended to aid your recovery and restore strength and mobility. 7. Long-term follow-up: After the surgery, regular follow-up visits with your healthcare provider will be necessary to monitor your progress and assess the success of the bypass. It's essential to attend these appointments and report any concerns or changes in your symptoms. 8. Lifestyle changes: Femoro-popliteal bypass surgery is not a cure for the underlying condition. To maintain the benefits of the procedure and reduce the risk of future complications, lifestyle modifications are crucial. This may involve quitting smoking, adopting a healthy diet, exercising regularly, and managing other risk factors such as high blood pressure, diabetes, or high cholesterol. It is important to have a thorough discussion with your healthcare provider about the procedure, your specific condition, and any concerns you may have. They will be able to provide personalized information and guidance to help you make an informed decision about whether femoro-popliteal bypass surgery is the right option for you.

Appendix D

Tests of Normality 

**Table 3:** Tests of normality using the Kolmogorov-Smirnov test of normality KS: Kolmogorov-Smirnov test statistic, (D): Lower numbers indicating a greater Gaussian distribution pattern FKRE: Flesch-Kincaid reading ease, FKGL: Flesch-Kincaid grade level, GFS: Gunning fog score, SMOG: Simple measure of gobbledygook

Readability scores	Mean	Median	SD	KS (D)	Gaussian?
FKRE	46.2	46.35	14.460982	0.24991	Yes
FKGL	11.08333	11.45	1.894642	0.29363	Yes
GFI	14.71667	15.15	2.249815	0.30739	Yes
SMOG	10.8	11.1	1.667333	0.28477	Yes
DISCERN	52.75714	52.5	3.375824	.21269	Yes
